# Avatar customization, social presence, and eHealth literacy: understanding user adoption of virtual hospitals for public health innovation

**DOI:** 10.3389/fpubh.2025.1706897

**Published:** 2025-12-18

**Authors:** Haoran Li, Sijie Sun, Zike Jing, Ruijie Zhang, Yilu He

**Affiliations:** 1 Department of Philosophy, Autonomous University of Barcelona, Barcelona, Spain; 2 College of Architecture and Art, Taiyuan University of Technology, Taiyuan, China; 3 College of Arts and Media, Kunming University of Science and Technology, Kunming, Yunnan, China; 4 School of Fine Arts, Shanxi Normal University, Taiyuan, China; 5 City University of Macau, Taipa, Macao SAR, China

**Keywords:** virtual hospital, avatar customization, social presence, eHealth literacy, metaverse healthcare services

## Abstract

**Introduction:**

Global healthcare systems face escalating challenges due to an aging population, an increasing illness burden, and significant shortages in the healthcare workforce. In this context, rapid advances in digitalisation and metaverse technologies have positioned virtual hospitals as a potentially transformative solution. However, research has largely focused on technological and clinical implementation, leaving open the question of how avatar-based design features and users’ eHealth literacy jointly shape psychological experiences and adoption behaviors in virtual hospital environments. This study addressed this gap by examining whether avatar customisation, avatar identification, social presence, and eHealth literacy influence users’ intention to use virtual hospitals.

**Methods:**

An online experiment was conducted with 415 participants recruited from an online panel. Participants were exposed to a virtual hospital scenario featuring varying levels of avatar customisation and then completed validated measures of avatar identification, social presence, eHealth literacy, and intention to use the virtual hospital.

**Results:**

Avatar customisation significantly increased users’ intention to use the virtual hospital by enhancing their sense of presence and engagement. Avatar identification and social presence operated as sequential mediators: higher avatar customisation strengthened avatar identification, which in turn increased social presence, ultimately leading to greater usage intention. In addition, eHealth literacy moderated the effect of social presence on usage intention, such that this relationship was stronger among individuals with higher levels of eHealth literacy.

**Discussion:**

These findings suggest that avatar-based design and users’ eHealth literacy jointly shape key psychological processes underlying the adoption of virtual hospitals. The results are consistent with and extend prior work on avatar identification and social presence by demonstrating their combined mediating role in a virtual healthcare context. The study offers practical implications for designing virtual hospital systems that foster identification and social presence, and it highlights the importance of supporting users’ eHealth literacy. Future research could further explore these mechanisms in different cultural settings and with longitudinal designs.

## 1 Introduction

Global healthcare systems are under increasing strain due to escalating expenditures driven by population growth, technological advancements, and, more critically, an aging population coupled with the rising prevalence of chronic and comorbid diseases (1, 2). These challenges have intensified the demand for medical services. At the same time, widespread workforce shortages, particularly in the nursing profession, have heightened the urgency to deliver more care with fewer human resources (3, 4). The COVID-19 pandemic has further exacerbated these systemic issues, accelerating the global adoption of telemedicine and remote care modalities (5, 6). In response to these pressures, metaverse-based healthcare services have rapidly emerged as an innovative and potentially transformative approach to reimagining care delivery.

The global metaverse healthcare market reflects this growing momentum. In 2023, it was valued at USD 8.97 billion and is projected to grow at a compound annual growth rate of 49.3%, reaching USD 496.26 billion by 2033 (7). This explosive growth underscores the sector’s disruptive potential, particularly in the Asia-Pacific region, which is expected to experience the most rapid expansion (8). Metaverse technologies go beyond traditional telemedicine by enabling a wide range of applications, including immersive clinical simulations, virtual consultations, and data-driven patient management (6, 9, 10, 90). Moreover, the integration of blockchain and Internet of Things (IoT) technologies enhances interoperability among medical devices, wearables, and healthcare infrastructure. This convergence not only bridges online and offline services but also facilitates global virtual consultations, offering particular value to underserved or remote regions such as rural India (5, 11, 92).

Building on these technological foundations, the integration of metaverse, IoT, and blockchain has led to the development of immersive digital environments known as virtual hospitals (6, 12). Countries including the United States, China, the United Kingdom, Australia, and Israel have begun adopting virtual hospital models. For example, Mercy Hospital in the United States launched “Mercy Virtual” in 2015 to provide remote care to patients in rural Missouri. Similarly, the “RPA Virtual Hospital” in Sydney, Australia, delivers continuous care, allowing patients to receive hospital-level treatment from their homes. These real-world implementations illustrate the viability and scalability of virtual hospitals within diverse healthcare systems.

Despite these advances, much of the existing literature on virtual hospitals has primarily focused on technological architectures, clinical applications, and organizational models, while paying comparatively limited attention to users’ psychological experiences and behavioral mechanisms in these environments. In particular, little is known about how specific avatar-based design features, users’ identification with their avatars, and their eHealth literacy jointly shape social and experiential processes that drive adoption of virtual hospitals. Furthermore, prior research has rarely integrated these factors into a unified framework that explains how avatar customisation may influence usage intention through intervening psychological constructs and under what conditions these effects are strengthened or weakened (101).

At the core of virtual hospital functionality is the interaction between patients and healthcare providers through digital avatars, sometimes referred to as “digital twins,” rendered on metaverse platforms. Personalizing these avatars enhances users’ identification with their virtual representations and strengthens their sense of realism during digital interactions, thereby boosting engagement and behavioral intention (13, 14, 90). Avatar customization also enhances social presence, fostering a more immersive and interactive experience than traditional telehealth channels. This perceived presence plays a crucial role in improving outcomes in virtual healthcare delivery (15). Importantly, users’ behavioral intentions in virtual hospital environments are significantly influenced by their level of eHealth literacy. Individuals with higher eHealth literacy demonstrate greater proficiency in navigating digital health platforms, critically evaluating online health information, and making informed decisions in virtual care contexts (16, 17). This digital competence not only facilitates engagement but also reinforces social presence, ultimately increasing users’ willingness to adopt virtual healthcare services. However, empirical studies that simultaneously examine avatar customisation, avatar identification, social presence, and eHealth literacy within virtual hospital settings remain scarce, leaving important questions about their combined effects on users’ adoption behaviors insufficiently addressed (91).

In light of these considerations and research gaps, this study investigates how avatar customisation, avatar identification, social presence, and eHealth literacy interact to influence users’ behavioral intentions within virtual hospital environments. By explicitly focusing on these user-centered psychological mechanisms, the study advances beyond prior work that has emphasized mainly technological and clinical perspectives. Specifically, it proposes and tests a model in which avatar customisation affects intention to use virtual hospitals via sequential mediation through avatar identification and social presence, and examines how eHealth literacy moderates the link between social presence and usage intention. By systematically exploring these constructs and their interrelationships, the research aims to advance theoretical understanding of user experience in immersive digital healthcare settings and to offer actionable insights for the development and implementation of metaverse-enabled medical services.

## 2 Theoretical background

### 2.1 Metaverse-based virtual healthcare services

The healthcare sector has traditionally been cautious in adopting emerging technologies, primarily due to its inherently high-risk, high-cost nature and the presence of strict ethical and legal requirements surrounding patient privacy. However, recent years have seen growing optimism regarding the transformative potential of the metaverse in healthcare. The metaverse, defined as a computer-generated virtual environment that facilitates real-time user interaction (11, 18, 19), is built on four foundational technologies: digital twins, extended reality (XR), network infrastructure, and blockchain. Among these, XR (which includes virtual reality (VR), augmented reality (AR), mixed reality (MR), and other immersive interfaces) is widely considered a key enabler. By merging physical and digital realms, XR enables novel user experiences and supports the creation of new socio-technical ecosystems that underpin metaverse-based care delivery (20, 21).

Through this convergence of technologies, the metaverse is becoming increasingly capable of enhancing routine diagnostics and treatment delivery (2, 9), while offering advantages across a range of critical healthcare scenarios. These include surgical training for complex procedures such as coronary artery bypass grafting, rehabilitation therapy in high-precision controlled environments, increased healthcare access for underserved or remote areas, and the management of highly infectious diseases (22, 23). By integrating these technologies into immersive digital spaces, the metaverse is reshaping traditional healthcare paradigms, enabling more scalable, flexible, and accessible care delivery models.

**Figure 1:**
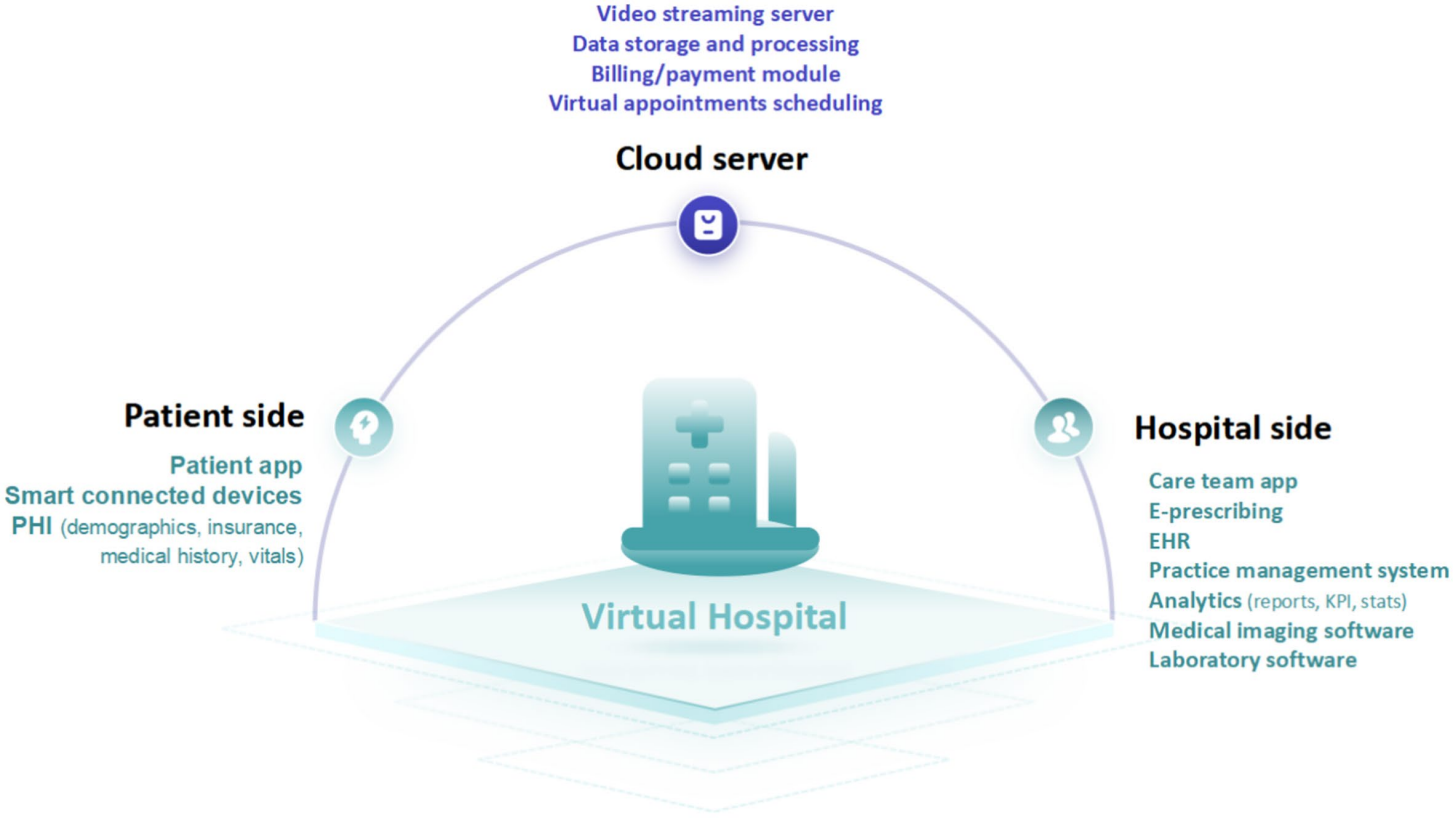
Three major blocks of virtual hospital.

Among these applications, virtual hospitals have received particular attention. These are comprehensive digital platforms designed to deliver a wide range of healthcare services online, thereby reducing the need for in-person visits and alleviating the burden on physical infrastructure (5, 24). A typical virtual hospital comprises three essential components: a patient-facing interface, a provider-side application, and cloud-based infrastructure (see Figure 1) (24, 25). Within this framework, patients receive continuous, home-based care resembling traditional hospital experiences, while healthcare providers access and analyze real-time patient data through interconnected devices. This model not only expands the reach and efficiency of care delivery but also improves patient engagement and accessibility, redefining the boundaries of conventional healthcare.

Although prior research has focused on the technological implementation and application of virtual hospitals (6, 12, 26), limited attention has been given to users’ psychological experiences and behavioral responses in these environments. This study addresses that gap by exploring the social, cognitive, and literacy-based mechanisms that shape user engagement in virtual hospital settings.

### 2.2 Avatar customization and intention to use virtual hospital

The term avatar originates from Sanskrit, originally referring to the descent of a deity into the human realm. In the metaverse context, avatars are widely understood as digital embodiments of the self, serving as visual and interactive representations of users within mediated environments (27, 28). In healthcare settings, a patient’s avatar functions as a digital proxy, enabling individuals to access medical services in virtual environments designed to replicate the look and feel of physical hospitals or clinics (11, 29). This function is often realized through digital twin technology, which produces data-driven replicas of real-world individuals.

Avatar customization refers to the process by which users design and select specific character attributes to personalize their digital representations (14, 30). These attributes commonly include skin tone, eye color, hairstyle, height, body shape, clothing, accessories, and, in some cases, personality traits. When users actively engage in this process, they participate in a form of digital self-expression. Prior studies have shown that individuals frequently design avatars to resemble their real-world appearance (27, 31), underscoring the link between avatar customization and self-concept through the externalization of digital identity.

The psychological mechanism underlying this phenomenon is often explained by the Proteus Effect, which posits that individuals’ behaviors and attitudes may align with the characteristics of their avatars (32). Grounded in self-perception theory, the Proteus Effect suggests that individuals form beliefs about themselves by observing their avatar-mediated behaviors from an external perspective (33). In virtual contexts, avatars function as embodied representations of the self, shaping self-perception and subsequently influencing user behavior (89). A stronger emotional bond with one’s avatar is associated with a more pronounced Proteus Effect (34, 35). Through customization, users reinforce identity cues by selecting features that reflect their physical or aspirational selves, thus enhancing the avatar–self connection.

Empirical research has consistently supported the positive outcomes of avatar customization across various digital contexts. It can help users focus on desirable attributes (27) and significantly increase system usage intention by fostering deeper immersion (30, 36). In healthcare-specific applications, avatar personalization has been found to strengthen emotional bonds between patients and their avatars, thereby improving communication and interaction with medical providers (37). This enhanced engagement not only boosts participation but also encourages treatment adherence (38). Moreover, in remote or privacy-sensitive scenarios, personalized avatars facilitate better information exchange and amplify the sense of presence, ultimately increasing users’ intention to seek healthcare services through virtual platforms (39, 95).

Based on the above discussion, the following hypothesis is proposed:

*H1*: Avatar customization positively influences users’ intention to use virtual hospital.

### 2.3 The mediating role of avatar identification

Avatar identification refers to the process by which users perceive themselves as their avatars for a certain period, establishing both emotional and cognitive connections with them (40, 41, 100). It is a psychological phenomenon in which individuals envision themselves as their avatars, enabling a deeper understanding of and empathy toward the avatar’s virtual context (42). To explain how avatars influence user behavior, Klimmt et al. (43) proposed the avatar identification theory, grounded in self-perception theory. This framework identifies four key dimensions of avatar identification: (1) emotional attachment, reflecting the affective bond between users and their avatars in virtual environments; (2) spatial presence or immersion, indicating the extent of user involvement; (3) positive evaluation, referring to users’ favorable assessment of the avatar’s performance or achievements; and (4) self–avatar merging, which involves the integration of the avatar’s traits and successes into the user’s self-concept (44, 45). In virtual hospital environments, avatars function as real-time digital proxies that represent patients’ identities, health data, and medical histories. Rather than being fictional characters, these avatars act as operational agents through which users receive diagnosis and treatment, thereby reinforcing their identification with the avatar (99).

Identification is generally strengthened by perceived similarity (97); users are more likely to identify with avatars that closely resemble their own physical or personal attributes (13, 46). Avatar customization facilitates this resemblance by allowing users to personalize avatars in ways that reflect their real-world identity, which in turn enhances identification. Numerous studies have demonstrated that avatar identification can significantly influence user behavior in virtual environments. For instance, in gaming contexts, higher levels of avatar identification are associated with stronger player loyalty and increased community participation (47). Similarly, Ko and Park (48) found that avatar identification positively affects users’ willingness to make purchases in virtual environments. In healthcare settings, users who identify with avatars that resemble themselves show stronger intentions to engage with health-related applications (14). Furthermore, the use of highly self-similar avatars in digital health interventions has been shown to enhance intervention effectiveness (49).

Based on these findings, the following hypothesis is proposed:

*H2*: Avatar identification mediates the relationship between avatar customization and intention to use virtual hospital.

### 2.4 The mediating role of social presence

The concept of social presence was first introduced by Short, Williams, and Christie (1976), who defined it as the degree to which individuals perceive others as being “real” within mediated communication. It refers to the extent to which users perceive the psychological, emotional, and intentional presence of others during technology-mediated interactions (50, 51). The social dimension of presence emphasizes warmth, personalization, emotional sensitivity (96), and the human quality of digital interaction [Animesh et al., 2011; (52)], with a primary focus on interpersonal communication in mediated environments (53).

Building on this foundational theory, Mennecke et al. (54) proposed the Embodied Social Presence Theory in response to advances in immersive virtual environments. This framework suggests that users experience social presence not only through the perceived presence of others but also through awareness of their own presence via avatars, fostering a shared sense of being together in avatar-mediated interactions.

Empirical studies have demonstrated that the realism of avatars significantly influences users’ perceived social presence. For instance, Garau et al. (55) and Dubosc et al. (15) found that avatars exhibiting high visual fidelity and lifelike gaze behavior enhance social presence by approximating real-world social cues. Similarly, Nowak and Biocca (56, 94) showed that more human-like avatars result in a stronger sense of social presence. Oh et al. (57, 102) further confirmed that avatars that closely resemble real people in both appearance and behavior foster deeper perceptions of realism and interpersonal connection. In virtual healthcare settings, enabling avatar customization to reflect users’ identity and preferences can enhance the perceived authenticity of clinical interactions.

Social presence has been shown to play a significant role in shaping users’ intentions to adopt virtual healthcare services. Higher levels of social presence foster a stronger sense of interpersonal connection and engagement, which in turn promotes greater willingness to participate in virtual hospital environments (58, 59). As a key experiential determinant, social presence contributes to higher satisfaction and stronger behavioral intentions (60, 61). Enhancing social presence through avatar customization may thus serve as an effective strategy for increasing the adoption of virtual healthcare systems. Based on these findings, the following hypothesis is proposed:

*H3*: Social presence mediates the positive relationship between avatar customization and users’ intention to use virtual hospital.

Furthermore, users who identify strongly with their avatars are more likely to experience heightened social presence, as identification promotes social identity and emotional bonding in digital spaces (47, 62). These dynamics foster perceived social support and interpersonal connection (52, 63, 64).

*H4*: Avatar identification and social presence sequentially mediate the effect of avatar customization on users’ intention to use virtual hospitals.

### 2.5 The moderating role of eHealth literacy

eHealth literacy refers to an individual’s ability to seek, access, comprehend, evaluate, and apply health information obtained through digital sources in order to make informed health decisions (65–67). Individuals with higher levels of eHealth literacy are typically better equipped to address personal health concerns through the effective use of digital platforms. This multidimensional construct comprises six core competencies: traditional literacy, information literacy, media literacy, health literacy, computer literacy, and scientific literacy (59, 68, 93).

While the majority of smartphone users engage in online searches for health-related information or medical advice, the internet is saturated with misinformation, disinformation, and low-quality health content (69, 70). As such, the ability to critically assess the credibility and relevance of digital health information is essential for responsible health decision-making in digital contexts.

In virtual environments, eHealth literacy has been shown to play an important role in shaping user engagement. Individuals with higher levels of eHealth literacy tend to navigate virtual platforms more effectively, decode social cues more accurately, and engage more meaningfully with digital healthcare systems. These skills directly contribute to a stronger sense of social presence (71, 72, 103). Furthermore, previous research has indicated that eHealth literacy significantly shapes users’ perceptions of the effectiveness, usability, and trustworthiness of digital health services. Individuals with high eHealth literacy are also more likely to adopt favorable attitudes toward emerging healthcare technologies and to perceive such tools as beneficial for improving health outcomes (17).

Several studies have further confirmed the moderating role of eHealth literacy in technology adoption behaviors. For instance, it has been found to moderate the relationships between internet access, technology use, and engagement with eHealth services (73). Given its proven behavioral relevance in prior studies, the present research proposes the following hypothesis:

*H5*: eHealth literacy positively moderates the effect of social presence on users’ intention to use virtual hospital.

## 3 Research methodology

### 3.1 Research model

**Figure 2:**
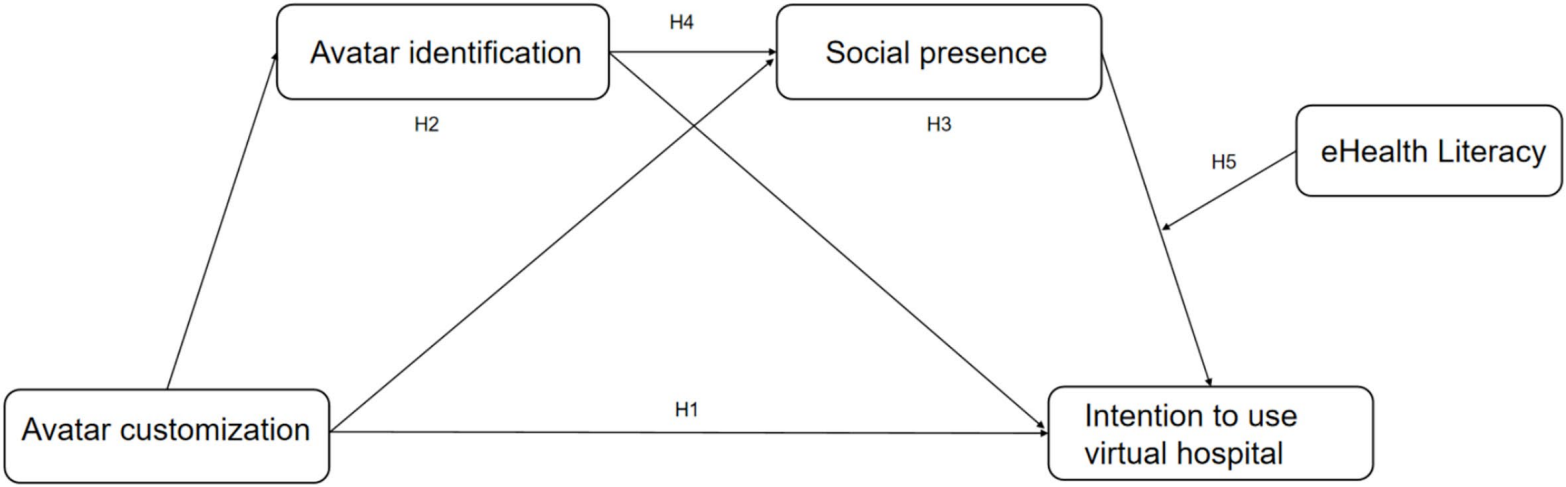
Research model.

To better understand the psychological mechanisms underlying virtual healthcare adoption, this study investigates the direct and indirect effects of avatar customization on users’ intention to use virtual hospital. Specifically, it examines avatar identification and social presence as sequential mediators, and eHealth literacy as a moderator. The conceptual model guiding this investigation is illustrated in Figure 2.

### 3.2 Participants

Participants in this study were recruited through the participant database of Credamo, a professional Chinese online survey platform^1^, and received monetary compensation for their involvement. To ensure the appropriateness of the sample, eligible participants were required to be adults (aged 18 years or older) and to have prior experience using remote services. Ethical approval was obtained from the Ethics Committee of the Autonomous University of Barcelona (Approval Number: 20241115CSH).

Prior to participation, all respondents were informed that they could raise questions regarding any part of the survey and were free to withdraw from the study at any time without penalty, and all participants provided informed consent. Following data screening and the exclusion of invalid responses, a total of 415 valid adult participants completed the experiment between January 6 and January 10, 2025. Demographic details are provided in Table 1. Most respondents were relatively young adults: 67.5% were between 18 and 40 years old, indicating that the sample is somewhat skewed toward younger users who are typically more familiar with digital technologies and remote services.

**Table 1:** Demographic information of participants (*N* = 415).

Category	Demographic characteristics	Number of participants	Percentage (%)
Gender	Male	217	52.3
	Female	198	47.7
Age	18–30	158	38.1
	31–40	122	29.4
	41–50	78	18.8
	51–60	40	9.6
	61+	17	4.1
Education	Elementary school	46	11.1
	Middle school	93	22.4
	High school	169	40.7
	University	107	25.8

### 3.3 Research design

**Figure 3:**
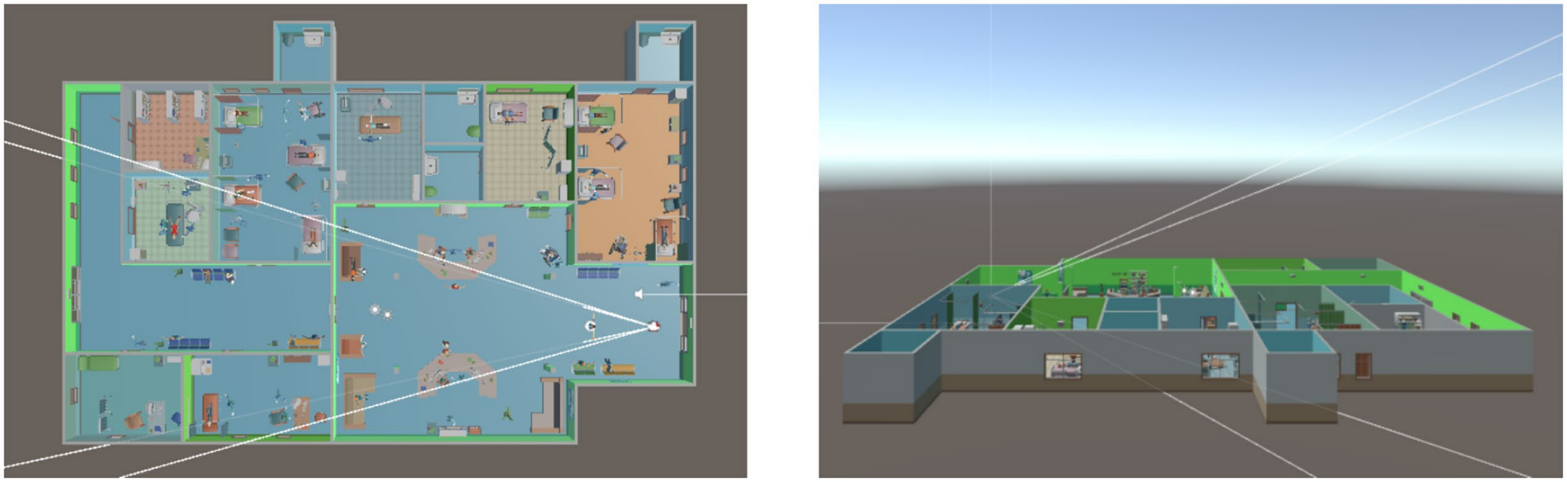
Virtual hospital model in unity.

This study aims to comprehensively investigate the mechanisms through which avatar customization influences users’ intention to use virtual hospitals. Specifically, it examines the mediating roles of avatar identification and social presence, as well as the moderating role of eHealth literacy. The study was conducted via an online experiment using a virtual hospital platform developed with the Unity (see Figure 3).

**Figure 4:**
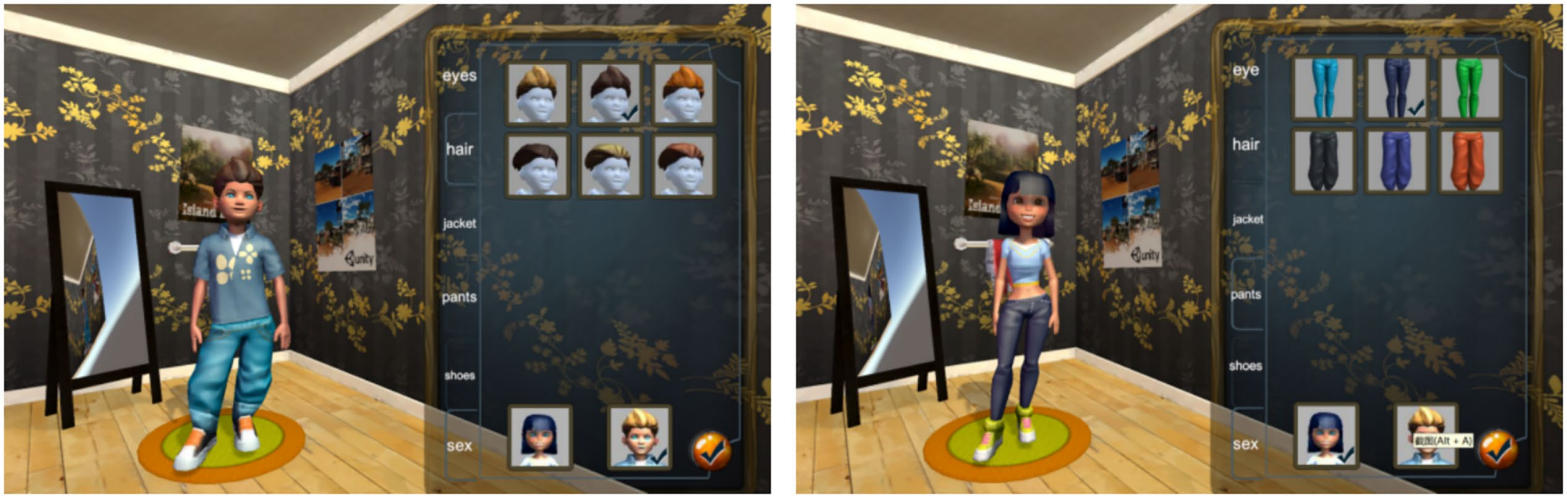
Avatar customization interface.

**Figure 5:**
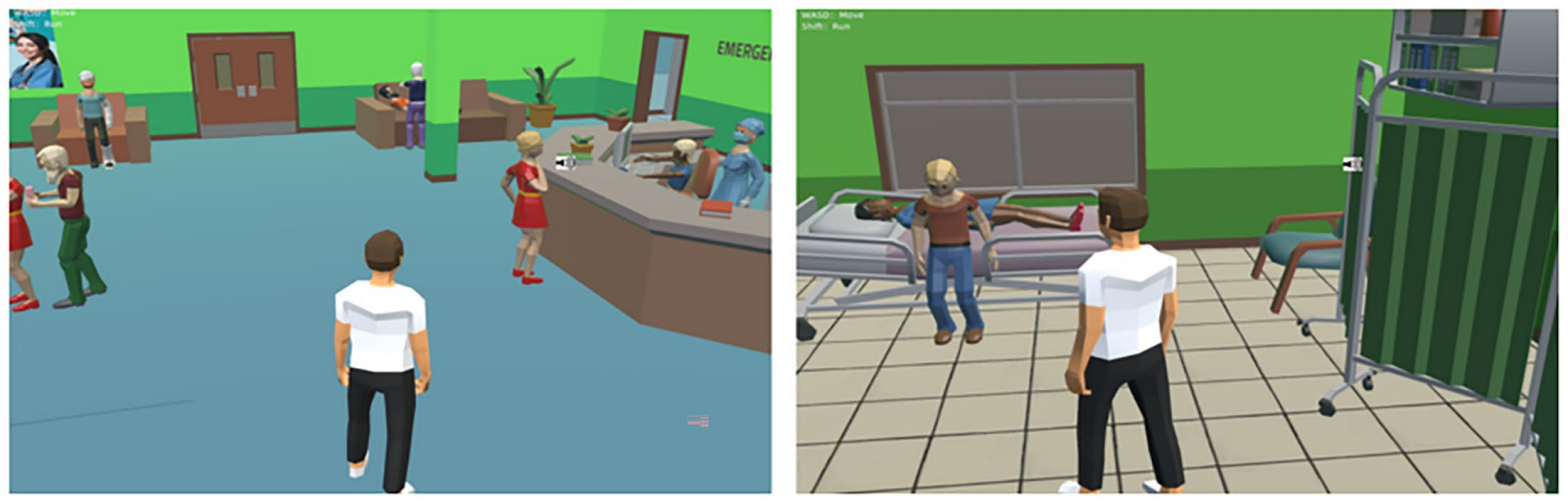
Interior view of the virtual hospital.

The system includes multiple functional areas, such as a reception desk, inpatient wards, consultation rooms, and operating rooms, designed to closely replicate the layout and interior structure of a real-world hospital (see Figures 4, 5).

A total of 415 participants were recruited and participated remotely in the experiment. Each participant accessed the virtual hospital system by logging in through a link provided via the Credamo platform. Upon entry, participants first engaged in avatar customization using the system’s built-in features. They were allowed to adjust various visual attributes of their avatars based on personal preferences, including gender, facial features, and clothing. After completing the customization process, participants explored the hospital’s virtual functions and underwent a simulated medical consultation. This process was designed to enhance their sense of avatar identification and perceived social presence within the hospital environment. Following the simulation, participants completed an online questionnaire measuring the study’s focal variables.

Avatar customization was measured using four items, such as: “I am able to customize my avatar as I wish” and “Customizing my avatar greatly enhances my immersion in the hospital experience” (42, 74). Avatar identification was assessed using six items, including: “My avatar is a representation of myself” and “When I use the virtual hospital, I feel that I merge with my avatar” (41). Social presence was measured using five items based on the work of Andel et al. (2020), such as: “There is a sense of sociality when using the virtual hospital” (1 = strongly disagree, 5 = strongly agree, *α* = 0.941) (Andel et al., 2020) (41). eHealth literacy was measured using five items, including: “I know how to use the information I find in the virtual hospital to help me” and “I know what resources are available within the virtual hospital”. All measurement items were rated on a 5-point Likert scale (1 = strongly disagree, 5 = strongly agree). Intention to use the virtual hospital was measured using four items, such as: “I have the skills I need to evaluate the resources I find in the virtual hospital” (Emmert and Wiener, 2017; Liu and Shi, 2021) (37).

To analyze the data, structural equation modeling (SEM) was employed to test the direct effect of avatar customization on intention to use the virtual hospital, as well as the mediating roles of avatar identification and social presence. In addition, moderation analysis was conducted to examine the moderating effect of eHealth literacy on the relationship between social presence and usage intention. Confirmatory factor analysis (CFA) and SEM were also performed to assess the reliability and validity of the measurement instruments. The results showed that all items yielded acceptable factor loadings and standardized estimates, indicating that the scales used in this study demonstrated strong psychometric properties in measuring various dimensions of users’ experiences in virtual hospital environments.

### 3.4 Measurement

This study assessed five key variables: avatar customization, avatar identification, social presence, intention to use the virtual hospital, and eHealth literacy. Table 2 outlines the specific measurement items corresponding to each construct. To evaluate the reliability and validity of the measurement instruments, confirmatory factor analysis (CFA) and structural equation modeling (SEM) were conducted.

**Table 2:** Measurement items and their factor loadings.

Study measures		Measurement items	CFA	SEM
Avatar customization	AC1	I am able to customize my avatar as I wish.	0.737	0.737
	AC2	Customizing my avatar greatly enhances my immersion in the hospital experience.	0.756	0.756
	AC3	Customizing my avatar is related to my use of this hospital.	0.758	0.758
	AC4	I can customize my avatar the way I want.	0.758	0.758
Avatar identification	AI1	My avatar resembles me.	0.8	0.8
	AI2	My avatar is a representation of myself.	0.717	0.717
	AI3	When I use the virtual hospital, I feel that I merge with my avatar.	0.814	0.814
	AI4	When using the virtual hospital, I feel that my avatar’s body becomes my body.	0.78	0.78
	AI5	My avatar is my role model.	0.851	0.851
Social presence	SP1	There is a sense of human contact when using the virtual hospital.	0.773	0.773
	SP2	There is a perception of personal individuality when using the virtual hospital.	0.753	0.753
	SP3	There is a sense of sociality when using the virtual hospital.	0.722	0.722
	SP4	There is a sense of warmth when using the virtual hospital.	0.744	0.744
	SP15	There is a sense of human sensitivity when using the virtual hospital.	0.747	0.747
Intention to use virtual hospital	IU1	I intend to use the virtual hospital the next time I need health services.	0.771	0.771
	IU2	Using the virtual hospital for remote treatment is a good option.	0.782	0.782
	IU3	If I become ill, I will choose a virtual hospital for an online consultation.	0.805	0.805
	IU4	If people around me need health services or remote treatment, I will recommend the virtual hospital to them.	0.789	0.789
eHealth literacy	eHL1	I know what resources are available within the virtual hospital.	1.000	1.000
	eHL2	I know where to find helpful resources within the virtual hospital.	0.769	0.769
	eHL3	I know how to use the information I find in the virtual hospital to help me.	0.914	0.914
	eHL4	I have the skills I need to evaluate the resources I find in the virtual hospital.	0.904	0.904
	eHL5	I feel confident in using information from the virtual hospital to make health decisions.	0.669	0.669

The results indicated that all factor loadings and standardized estimates met acceptable thresholds, confirming the strong psychometric properties of the measurement scales used in this study. For instance, within the avatar customization construct, the item “I am able to customize my avatar as I wish” demonstrated a factor loading of 0.737, indicating a strong representation of the latent variable. Similarly, for the social presence construct, the item “There is a sense of warmth when using the virtual hospital” yielded a factor loading of 0.744, further supporting the construct’s validity.

Overall, these results confirm that the measurement instruments exhibit high levels of reliability and validity, effectively capturing the multidimensional nature of user experiences within the virtual hospital environment.

## 4 Results analysis

### 4.1 Data analysis and model evaluation

#### 4.1.1 Normality and common method Bias

The skewness and kurtosis values for all variables fall within the acceptable range of ±1.96, indicating that the data are approximately normally distributed. The regression model is statistically significant (*F* = 105.887, *p* < 0.001), suggesting that the model has strong explanatory power. Further analysis shows that avatar customization (X), avatar identification (M1), and social presence (M2) all have significant effects on the intention to use virtual hospitals (Y), with all *p*-values less than 0.05.

Multicollinearity diagnostics indicate that all variance inflation factor (VIF) values are below 5 and all tolerance values exceed 0.10, suggesting no multicollinearity issues. Principal component analysis (PCA) conducted on 18 observed variables extracted four components, which collectively explain 68.605% of the total variance. This confirms the structural validity of the factor model.

#### 4.1.2 Reliability and validity

Composite reliability (CR) and average variance extracted (AVE) were used to assess the internal consistency and convergent validity of each construct. The CR values for avatar customization, avatar identification, social presence, and usage intention were 0.747, 0.843, 0.780, and 0.801, respectively, all exceeding the recommended threshold of 0.70. Additionally, all AVE values were above 0.50, indicating good convergent validity (see Table 3).

**Table 3:** Construct reliability and validity indicators.

Construct	CR	AVE	MSV	ASV	AC	AI	SP	IU
Avatar customization	0.747	0.566	0.324	0.193	1	0.569	0.435	0.254
Avatar identification	0.843	0.63	0.324	0.171	0.569	1	0.326	0.29
Social presence	0.780	0.559	0.189	0.132	0.435	0.326	1	0.316
Intention to use virtual hospital	0.801	0.619	0.1	0.083	0.254	0.29	0.316	1

**Figure 6:**
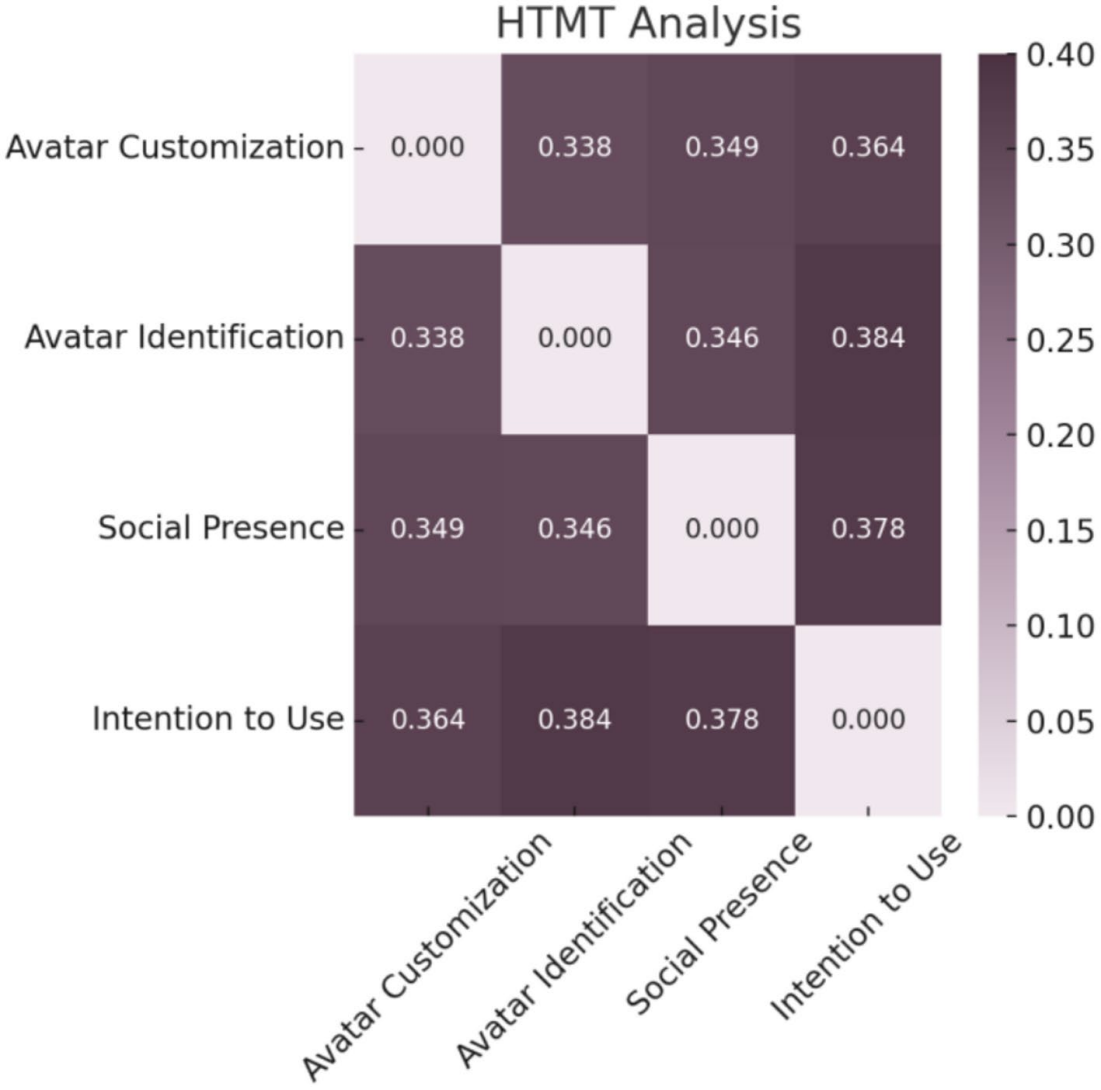
HTMT analysis.

Discriminant validity was supported by the fact that the AVE values of each construct exceeded their respective maximum shared variance (MSV), and all heterotrait–monotrait (HTMT) ratios were below 0.85 (see Figure 6). The model fit indices also demonstrate a strong model-data fit: *χ^2^/df* = 1.453, RMR = 0.024, GFI = 0.954, AGFI = 0.939, TLI = 0.983, CFI = 0.985, and RMSEA = 0.033 (see Table 4).

**Table 4:** Model fit indices summary.

CMIN/DF	RMR	GFI	AGFI	TLI	CFI	RMSEA
1.453	0.024	0.954	0.939	0.983	0.985	0.033
<3	<0.08	>0.85	>0.85	>0.9	>0.9	<0.08

### 4.2 Structural model analysis

The results of the path analysis indicate that avatar customization has a significant positive effect on the intention to use virtual hospitals, with a standardized regression coefficient of 0.254 (*p* < 0.05) (see Table 5). This suggests that users who engage in avatar customization are more likely to intend to use virtual healthcare services, thereby supporting Hypothesis H1.

**Table 5:** Hypothesis 1 test results.

Path	*β* (Estimate)	Significance	Support
H1: Avatar customization → Intention to use virtual hospital	0.254	<0.05	Yes

Moreover, the indirect effects of avatar customization, mediated through avatar identification and social presence, are also statistically significant. These findings highlight the importance of both cognitive and social mechanisms in explaining how avatar customization influences user behavior. The overall model demonstrates a good fit to the data, further validating the proposed structural relationships.

### 4.3 Mediation analysis

First, the results indicate that avatar customization (X) has a significant positive effect on avatar identification (M1), with a standardized regression coefficient of 0.4693 and a p-value less than 0.001 (see Table 6). This suggests that users who engage in avatar customization are more likely to develop identification with and emotional connection to their avatars. In addition, avatar customization (X) also directly influences the intention to use virtual hospitals (Y), with a coefficient of 0.3581 (*p* < 0.001), indicating that customization can effectively enhance users’ willingness to adopt virtual healthcare services. Avatar identification (M1) itself also exerts a significant impact on usage intention (Y), with a coefficient of 0.4124 (*p* < 0.001). Mediation analysis further reveals that the indirect effect of X on Y through M1 is 0.1935. Taken together, these findings provide empirical support for Hypotheses H1 and H2, demonstrating the direct and mediated influence of avatar customization on user intention.

**Table 6:** Mediation effect analysis results.

Hypothesis	Parameter	Coefficient	SE	*t*	*p*	LLCI	ULCI
H2	Avatar Customization (X) → Avatar Identification (M1)	0.4693	0.0408	11.4905	0.000	0.389	0.5496
	Avatar Customization (X) → Intention to Use (Y)	0.3581	0.0466	7.6839	0.000	0.2665	0.4497
	Avatar Identification (M1) → Intention to Use (Y)	0.4124	0.0489	8.4366	0.000	0.3163	0.5084
	Indirect Effect of X on Y through M1	0.1935	0.0441	-	-	0.1178	0.2899
H3	Avatar Customization (X) → Social Presence (M2)	0.478	0.0377	12.6639	0.000	0.4038	0.5522
	Social Presence (M2) → Intention to Use (Y)	0.447	0.0529	8.454	0.000	0.343	0.5509
	Indirect Effect of X on Y through M2	0.2136	0.0464	-	-	0.1321	0.3135
H4	Avatar Customization (X) → Avatar Identification (M1)	0.4693	0.0408	11.4905	0.000	0.389	0.5496
	Avatar Customization (X) → Social Presence (M2)	0.3302	0.0408	8.0902	0.000	0.25	0.4104
	Avatar Identification (M1) → Social Presence (M2)	0.3149	0.0428	7.3575	0.000	0.2308	0.3991
	Indirect Effect of X on Y through M1 and M2	0.0493	0.0185	-	-	0.0195	0.0914

Second, the analysis shows that avatar customization (X) significantly enhances users’ perceived social presence (M2), with a coefficient of 0.478 (*p* < 0.001), suggesting that customized avatars increase users’ sense of interpersonal connection within virtual environments. Social presence (M2) also has a strong positive effect on intention to use virtual hospitals (Y), with a coefficient of 0.447 (*p* < 0.001). The indirect effect of X on Y via M2 is 0.2136, indicating that social presence serves as a key psychological pathway in promoting virtual healthcare adoption. These results confirm Hypothesis H3.

Furthermore, the extended mediation model demonstrates that avatar customization (X) significantly influences both avatar identification (M1) and social presence (M2), and that avatar identification (M1) also positively affects social presence (M2). Specifically, the effect of X on M1 is 0.4693 (*p* < 0.001), the effect of X on M2 is 0.3302 (p < 0.001), and the effect of M1 on M2 is 0.3149 (*p* < 0.001) (see Table 5). The sequential mediation analysis reveals a combined indirect effect of X on Y through M1 and M2 of 0.0493. This suggests that avatar customization enhances users’ intention to use virtual hospitals by sequentially increasing their avatar identification and perceived social presence. These findings provide strong support for Hypothesis H4.

### 4.4 Moderating effect of eHealth literacy

Using Model 1 of the PROCESS macro and performing bootstrap analysis with 5,000 resamples, the results indicate that eHealth literacy significantly moderates the relationship between social presence and the intention to use virtual hospitals (see Table 7). Specifically, the positive association between social presence and usage intention becomes stronger when individuals possess a higher level of eHealth literacy.

**Table 7:** Mediation effect analysis results.

Parameter	Coeff	SE	*t*	*p*	LLCI	ULCI
Constant	2.7977	0.5994	4.6676	0.0000	1.6195	3.9760
Social presence	0.1958	0.1432	1.3676	0.1722	−0.0857	0.4774
eHealth literacy	−0.4199	0.1755	−2.3924	0.0172	−0.7649	−0.0749
Interaction	0.1322	0.0415	3.1858	0.0016	0.0506	0.2137

**Figure 7:**
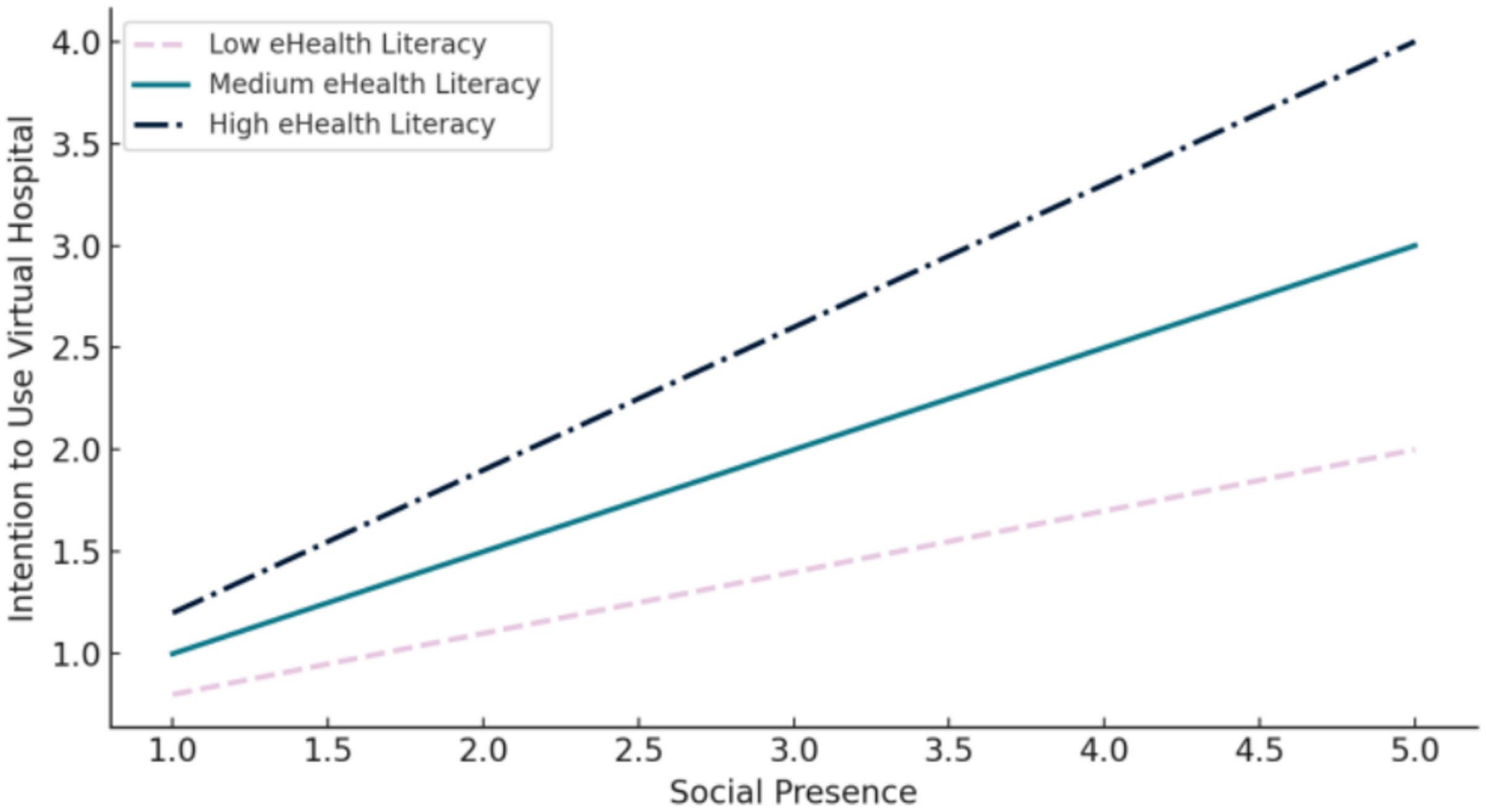
Interaction effect of eHL on the relationship between SP and IU.

Moreover, the moderation analysis shows that the interaction term between social presence and eHealth literacy yields a statistically significant regression coefficient of 0.1322 (*p* = 0.0016). This finding provides further evidence that eHealth literacy plays a meaningful moderating role in shaping the impact of social presence on users’ behavioral intentions (see Figure 7). In particular, when eHealth literacy is low, the influence of social presence on intention to use virtual hospitals is relatively weak. Conversely, when eHealth literacy is high, the effect becomes substantially stronger, highlighting the importance of digital health competencies in virtual healthcare adoption.

In conclusion, eHealth literacy significantly moderates the relationship between social presence and the intention to use virtual hospitals. As the level of eHealth literacy increases, the positive effect of social presence on usage intention becomes more pronounced, thereby supporting Hypothesis H5.

## 5 Discussion

This study explores the impact of avatar customization on user experience and the intention to adopt virtual hospitals. Based on empirical evidence, several direct, mediating, and moderating pathways were identified, offering both theoretical insights and practical implications.

First, the findings clearly indicate that personalized avatar customization significantly enhances users’ intention to use virtual hospitals. As digital self-representations, avatars promote immersion and self-expression, which increases user engagement in virtual healthcare environments. This personalized embodiment helps form a stronger sense of identity and psychological connection within the virtual space, ultimately reinforcing behavioral intention. These results are consistent with the work of Sestino et al. (37), who emphasized the importance of personalized digital identities in improving user engagement and platform retention. Avatars also act as communication tools, enabling users to convey health-related needs and emotions. This enhances the sense of presence and authenticity in the absence of physical interaction, as supported by Shinbane and Saxon (75) and Yu et al. (39). Therefore, future virtual healthcare systems should prioritize the development of robust avatar customization features to improve user experiences and promote wider adoption.

Second, grounded in social presence theory, which posits that technology-mediated environments can convey a sense of “being with others” comparable to face-to-face interaction (51, 53), the study confirms that social presence mediates the relationship between avatar customization and usage intention. Avatar identification is identified as a key factor that precedes social presence. When users are able to customize and relate to their avatars, they are more likely to feel a genuine sense of being in the virtual environment, which in turn enhances their intention to use virtual hospitals. The proposed sequential mediation model receives strong empirical support. This pattern is consistent with prior research showing that higher perceived social presence strengthens users’ trust, engagement, and technology acceptance in digital and e-health settings (52, 76). This finding is in line with Waddell et al. (14), who found that strong avatar identification contributes to positive behavioral intentions in virtual health contexts. Our study extends this view by demonstrating that avatar identification not only has a direct effect on usage intention but also exerts an indirect influence through increased social presence. This is further supported by Kim et al. (59), who noted that lifelike avatars encourage deeper personal connections and higher levels of engagement in virtual spaces.

In addition, the study confirms a sequential mediation effect linking avatar customization to usage intention through avatar identification and social presence. This reveals an important psychological mechanism by which personalized digital embodiment enhances user engagement and promotes behavioral outcomes within virtual healthcare platforms. These results echo the findings of Ching-I Teng (47), who showed that stronger avatar identification enhances social presence and, in turn, fosters user loyalty and continued platform use. This suggests that avatar customization should not be viewed merely as a visual or esthetic feature. Instead, it plays a vital role in supporting users’ psychological connection and long-term engagement with virtual health services.

Beyond these psychological mechanisms, the demographic composition of the sample helps contextualize the findings. Most respondents were relatively young adults: 67.5% were between 18 and 40 years old. Younger individuals are typically more open to adopting new digital technologies and engaging in eHealth behaviors (17), which may partly explain the generally high intention to use virtual hospitals observed in this study. At the same time, the under-representation of older adults suggests that the strength of the observed effects may differ in populations with lower technology readiness, a point that future research should examine more closely.

Finally, the study highlights the moderating role of eHealth literacy in the relationship between social presence and usage intention. Users with higher eHealth literacy are better equipped to understand, trust, and navigate virtual healthcare environments, which strengthens the impact of social presence on their intention to use such services. This underscores the importance of cognitive capabilities in digital health adoption. Jo et al. (77) observed that eHealth literacy is closely associated with self-efficacy and active information-seeking behavior, both of which are critical for achieving positive health outcomes. Alviani et al. (78) further noted that eHealth literacy helps users interpret and apply health information, enhancing not only individual health outcomes but also the acceptance and continued use of virtual medical platforms. Improving eHealth literacy should therefore be seen as a strategic priority in supporting user participation, reducing digital inequalities, and fostering the long-term development of virtual hospitals.

In conclusion, this study proposes and validates a theoretical model that connects avatar customization, avatar identification, social presence, and usage intention, with eHealth literacy as a key moderating factor. It contributes to a deeper understanding of user behavior in virtual healthcare settings, enriches the theoretical discussion on avatar-based interaction, and provides practical guidance for the development of future digital health platforms. By jointly modeling avatar-based design, socio-emotional experiences, and eHealth literacy in a virtual hospital context, the study offers an integrated perspective that has been largely absent from prior work, thereby advancing current understanding of how digitally mediated self-representation and cognitive capacities shape virtual healthcare adoption.

## 6 Theoretical and practical implications, and research limitations

### 6.1 Theoretical implications

This study provides several theoretical contributions to media psychology, digital health, and avatar-based interaction research. It offers valuable insights into users’ psychological engagement and behavioral patterns within virtual hospital environments. First, by showing that both avatar customization and avatar identification positively influence users’ intention to adopt virtual healthcare services, the study extends prior avatar research, which has traditionally focused on entertainment, gaming, and social media contexts, into a high stakes, utilitarian setting where health and well-being are at stake. This finding broadens the application of avatar-related research from its traditional focus on entertainment and social media to the field of digital health. It also contributes to a deeper understanding of identity formation in virtual contexts. Rather than viewing avatars purely through an esthetic or recreational lens, this study emphasizes their functional and symbolic importance in healthcare settings, offering a more comprehensive foundation for examining user-avatar interaction. In doing so, it demonstrates that identity-based mechanisms triggered by digital self-representation are not confined to hedonic environments but also shape behavior in serious, health-related domains.

Second, the study establishes and empirically supports a sequential mediation mechanism in which avatar customization influences usage intention through avatar identification and, subsequently, social presence. This supports the relevance of social presence theory in digital healthcare and illustrates how emotional and interpersonal experiences, commonly associated with in-person interactions, can be effectively recreated in virtual environments through well-designed digital agents (51, 53, 64). Whereas previous research has often examined avatar identification and social presence separately, the present study integrates them into a single process model, clarifying how design features at the interface level cascade through psychological experience to shape behavioral intention. The findings enhance existing frameworks by showing how a heightened sense of presence can strengthen users’ psychological connection with virtual health platforms and by explicating the pathway through which avatar-based design features translate into adoption-related outcomes.

In addition, this research is among the first to investigate the moderating effect of eHealth literacy on the association between social presence and behavioral intention in a virtual hospital context. The results indicate that users with higher levels of eHealth literacy are more capable of interpreting and responding to socio-emotional cues in virtual healthcare settings. This strengthens the effect of social presence on their intention to engage with virtual services. By introducing eHealth literacy as a boundary condition, the study adds a cognitive and informational competence perspective to models of technology acceptance and digital health adoption. It shows that socio-emotional experience and cognitive capacity jointly determine how users translate virtual interactions into concrete intentions to use virtual hospitals. These insights underscore the role of informational competence in shaping user responses within virtual ecosystems and connect the avatar and social presence literature with the broader stream of research on health literacy.

By proposing and validating a comprehensive analytical framework that integrates avatar-based design, social dynamics, and individual cognitive capacities, this study advances theoretical understanding of user behavior in complex digital health environments. It establishes a foundation for future research on digitally mediated health decision-making and user engagement in virtual care platforms. Taken together, these contributions demonstrate that avatar-based self-representation, perceived social presence, and eHealth literacy are interlocking mechanisms that help explain when and for whom virtual hospitals are likely to be adopted, thereby advancing theoretical debates on user experience and technology acceptance in metaverse-enabled healthcare.

### 6.2 Practical implications

This study offers practical recommendations for the design and implementation of virtual hospitals and other digital healthcare platforms.

First, platform developers should provide a wide range of meaningful avatar customization options. These should allow users to personalize not only visual characteristics such as appearance and clothing, but also behavioral features including voice, facial expressions, and modes of interaction. As highlighted by Oh Kruzic et al. (79) and Zempo et al. (80), these personalization features go beyond esthetics. They serve as tools for enhancing users’ sense of identity, thereby fostering deeper psychological engagement and a stronger emotional connection to the virtual environment.

Second, to strengthen the sense of social presence, virtual hospitals should be designed as immersive, interactive, and emotionally engaging spaces. Creating opportunities for authentic interpersonal interaction can increase users’ feelings of trust and satisfaction, which are critical for sustained engagement and long-term use of digital health services.

Furthermore, in light of the significant moderating role of eHealth literacy, virtual hospitals should incorporate tailored educational and training programs. These initiatives can help users develop the necessary skills to effectively navigate digital health systems and confidently participate in virtual care. During onboarding or marketing efforts, it is essential to implement differentiated support strategies based on users’ levels of eHealth literacy. As suggested by Ezeudoka and Fan (16) and Ukaegbu and Mingyue (81), such targeted approaches can enhance accessibility, encourage user participation, and reduce barriers to engagement. These efforts are especially important for addressing digital inequalities and promoting inclusive access to virtual healthcare services.

### 6.3 Limitations and directions for future research

Despite offering valuable insights, this study has several limitations that suggest promising directions for future research.

First, the avatar customization examined in this study was based on a single artistic style. Future research could investigate how varying visual styles, such as cartoon-like, hyper-realistic, or abstract designs, influence user experience, identity expression, and engagement. As noted by Kim et al. (59) and Takano and Taka (31), these stylistic differences may align with users’ cultural identities, esthetic preferences, or psychological needs. Exploring how visual design choices shape digital self-representation and emotional resonance could provide deeper insight into optimizing avatar design in virtual environments (82).

Second, this study focused exclusively on a virtual hospital context. While this approach yields targeted insights, the findings may not be fully applicable to other virtual domains such as education, gaming, or social networking. Future studies should assess whether the mechanisms observed here can be replicated across diverse virtual platforms to evaluate the broader applicability of avatar-related effects on user behavior (83).

Third, the data were collected from a single online panel in one country. Although this sampling strategy ensured consistency in data collection, it may limit the generalisability of the findings to other cultural, healthcare, or technological contexts. Future research should therefore replicate the proposed model with more diverse and cross-cultural samples to examine whether the observed relationships hold for broader populations.

Fourth, although eHealth literacy is theoretically multidimensional, comprising several distinct skills such as accessing, understanding, evaluating, and applying digital health information, the study treated it as a unidimensional construct for analytical simplicity. Future research could disaggregate eHealth literacy into its six core dimensions to gain a more detailed understanding of its influence. Additionally, other moderating variables such as health anxiety, gender-related stress, or privacy concerns regarding medical data should be considered. Prior studies, including those by Lebel et al. (84), Prettyman and Bolls (85), and Zahedi et al. (29), have emphasized the importance of these individual differences in shaping users’ digital health behaviors and interactions.

Finally, the study employed a cross-sectional design, and the data were collected at a single time point. As a result, the findings represent associations rather than definite causal relationships. Although there is increasing global interest in applying metaverse technologies to healthcare, real-world implementation remains limited. Most current efforts are confined to small-scale pilot projects with short-term evaluations. Future research should therefore employ longitudinal or multi-wave designs to examine how avatar customization, social presence, and other factors influence user intentions and health outcomes over time. Long-term studies would provide greater clarity on causal relationships and offer stronger evidence for the sustainable development and scaling of virtual healthcare platforms.

## Data Availability

The raw data supporting the conclusions of this article will be made available by the authors, without undue reservation.

## Footnotes

1
https://www.credamo.com/#/

## References

[1] Mathkor DM Mathkor N Bassfar Z Bantun F Slama P Ahmad F . Multirole of the internet of medical things (IoMT) in biomedical systems for managing smart healthcare systems: an overview of current and future innovative trends. J Infect Public Health. (2024) 17:559–72. doi: 10.1016/j.jiph.2024.01.013, 38367570

[2] Peters GM. Exploring the potential of virtual hospital care. Enschede: University of Twente (2024).

[3] Parzonka K Ndayishimiye C Domagała A. Methods and tools used to estimate the shortages of medical staff in european countries—scoping review. Int J Environ Res Public Health. (2023) 20:2945. doi: 10.3390/ijerph20042945, 36833641 PMC9957245

[4] Watson A. Health workforce shortages: do global healthcare dollars equate to workforce sense? J Perinat Neonatal Nurs. (2024) 38:124–5. doi: 10.1097/JPN.0000000000000811, 38758265

[5] Francis NA Stuart B Knight M Vancheeswaran R Oliver C Willcox M . Predictors of clinical deterioration in patients with suspected COVID-19 managed in a ‘virtual hospital'setting: a cohort study. BMJ Open. (2021) 11:e045356. doi: 10.1136/bmjopen-2020-045356, 33757955 PMC7992373

[6] Sitammagari K Murphy S Kowalkowski M Chou SH Sullivan M Taylor S . Insights from rapid deployment of a “virtual hospital” as standard care during the COVID-19 pandemic. Ann Intern Med. (2021) 174:192–9. doi: 10.7326/M20-4076, 33175567 PMC7711652

[7] Spherical Insights (2023) Global Metaverse in healthcare market insights forecasts to 2033. Available online at: https://www.sphericalinsights.com/reports/metaverse-in-healthcare-market (Accessed September 10, 2024).

[8] Vishwakarma LP Singh RK Mishra R Kumari A. Application of artificial intelligence for resilient and sustainable healthcare system: systematic literature review and future research directions. Int J Prod Res. (2025) 63:822–44. doi: 10.1080/00207543.2023.2188101

[9] Song YT Qin J. Metaverse and personal healthcare. Procedia Comput Sci. (2022) 210:189–97. doi: 10.1016/j.procs.2022.10.136

[10] Wang G Badal A Jia X Maltz JS Mueller K Myers KJ . Development of metaverse for intelligent healthcare. Nat Mach Intell. (2022) 4:922–9. doi: 10.1038/s42256-022-00549-6, 36935774 PMC10015955

[11] Tiwari A Dubey A Yadav AK Bhansali R Bagaria V. A review of smart future of healthcare in the digital age to improve quality of orthopaedic patient care in metaverse called: the Healthverse!! J Clin Orthop Trauma. (2024) 48:102340. doi: 10.1016/j.jcot.2024.102340, 38292151 PMC10823058

[12] Bidoli C Pegoraro V Dal Mas F Bagnoli C Bert F Bonin M . Virtual hospitals: the future of the healthcare system? An expert consensus. J Telemed Telecare. (2025) 31:121–33. doi: 10.1177/1357633X231173006, 37226478

[13] Koulouris J. Jeffery Z. Best J. O'neill E. Lutteroth C. 2020). Me vs. super (wo) man: effects of customization and identification in a VR Exergame. In Proceedings of the 2020 CHI conference on human factors in computing systems (pp. 1–17) Honolulu, HI: ACM

[14] Waddell TF Sundar SS Auriemma J. Can customizing an avatar motivate exercise intentions and health behaviors among those with low health ideals? Cyberpsychol Behav Soc Netw. (2015) 18:687–90. doi: 10.1089/cyber.2014.0356, 26406804

[15] Dubosc C Gorisse G Christmann O Fleury S Poinsot K Richir S. Impact of avatar facial anthropomorphism on body ownership, attractiveness and social presence in collaborative tasks in immersive virtual environments. Comput Graph. (2021) 101:82–92. doi: 10.1016/j.cag.2021.08.011

[16] Ezeudoka BC Fan M. Determinants of behavioral intentions to use an E-pharmacy service: insights from TAM theory and the moderating influence of technological literacy. Res Soc Adm Pharm. (2024) 20:605–17. doi: 10.1016/j.sapharm.2024.03.007, 38531706

[17] Magsamen-Conrad K Dillon JM Billotte Verhoff C Faulkner SL. Online health-information seeking among older populations: family influences and the role of the medical professional. Health Commun. (2019) 34:859–71. doi: 10.1080/10410236.2018.1439265, 29474125 PMC6230499

[18] Jeong E Lee D. Metaverse applications in healthcare: opportunities and challenges. Serv Bus. (2025) 19:4. doi: 10.1007/s11628-024-00577-9

[19] Shah V Khang A. Metaverse-enabling IoT technology for a futuristic healthcare system In: Khang A, editor. Handbook of research on AI-based technologies and applications in the era of the Metaverse. Pennsylvania: IGI Global (2023). 165–73.

[20] Massetti M Chiariello GA. The metaverse in medicine. Eur Heart J Suppl. (2023) 25:B104–7. doi: 10.1093/eurheartjsupp/suad083, 37091647 PMC10120971

[21] Zhao Y Jiang J Chen Y Liu R Yang Y Xue X . Metaverse: perspectives from graphics, interactions and visualization. Vis Inform. (2022) 6:56–67. doi: 10.1016/j.visinf.2022.03.002

[22] Gupta K Mathur S. Metaverse: revolutionizing healthcare in a virtual realm In: Wason R Arora P Nand P Jain V Kukreja V, editors. Blockchain-enabled solutions for the pharmaceutical industry. New York: Wiley (2025). 529–43.

[23] Shao L Tang WEI Zhang Z Chen X. Medical metaverse: technologies, applications, challenges and future. J Mech Med Biol. (2023) 23:2350028. doi: 10.1142/S0219519423500288

[24] Itransition, (2023). Virtual hospital: key features, examples, and benefits of remote care. Available online at: https://www.itransition.com/healthcare/virtual-hospital (Accessed November 20, 2024).

[25] 10xDS Team, 2025. What is Virtual Healthcare: Its essence, categories, and solutions. Available online at: https://10xds.com/blog/what-is-virtual-healthcare/ (Accessed February 5, 2025).

[26] Rifino N Bersano A Padovani A Conti GM Cavallini A Colombo L . Virtual hospital and artificial intelligence: a first step towards the application of an innovative health system for the care of rare cerebrovascular diseases. Neurol Sci. (2024) 45:2087–95. doi: 10.1007/s10072-023-07206-9, 38017154

[27] Kang H Kim HK. My avatar and the affirmed self: psychological and persuasive implications of avatar customization. Comput Hum Behav. (2020) 112:106446. doi: 10.1016/j.chb.2020.106446

[28] Scarborough JK Bailenson JN. Avatar psychology In: Grimshaw M, editor. The Oxford handbook of virtuality. Oxford: Oxford University Press (2014). 129–44.

[29] Zahedi FM Zhao H Sanvanson P Walia N Jain H Shaker R. My real avatar has a doctor appointment in the Wepital: a system for persistent, efficient, and ubiquitous medical care. Inf Manag. (2022) 59:103706. doi: 10.1016/j.im.2022.103706

[30] Ratan R Sah YJ. Leveling up on stereotype threat: the role of avatar customization and avatar embodiment. Comput Hum Behav. (2015) 50:367–74. doi: 10.1016/j.chb.2015.04.010

[31] Takano M Taka F. Fancy avatar identification and behaviors in the virtual world: preceding avatar customization and succeeding communication. Comput Human Behav Rep. (2022) 6:100176. doi: 10.1016/j.chbr.2022.100176

[32] Ratan R Klein MS Ucha CR Cherchiglia LL. Avatar customization orientation and undergraduate-course outcomes: actual-self avatars are better than ideal-self and future-self avatars. Comput Educ. (2022) 191:104643. doi: 10.1016/j.compedu.2022.104643

[33] Szolin K Kuss DJ Nuyens FM Griffiths MD. Exploring the user-avatar relationship in videogames: a systematic review of the Proteus effect. Hum Comput Interact. (2023) 38:374–99. doi: 10.1080/07370024.2022.2103419

[34] Bian Y Zhou C Tian Y Wang P Gao F. The proteus effect: influence of avatar appearance on social interaction in virtual environments In: Stephanidis C, editor. HCI International 2015 - Posters’ Extended Abstracts. HCI 2015. Communications in Computer and Information Science. Cham: Springer (2015)

[35] Ratan R Beyea D Li BJ Graciano L. Avatar characteristics induce users' behavioral conformity with small-to-medium effect sizes: a meta-analysis of the proteus effect. Media Psychol. (2020) 23:651–75. doi: 10.1080/15213269.2019.1623698

[36] Birk MV Mandryk RL. Improving the efficacy of cognitive training for digital mental health interventions through avatar customization: crowdsourced quasi-experimental study. J Med Internet Res. (2019) 21:e10133. doi: 10.2196/10133, 30622095 PMC6329434

[37] Sestino A D'Angelo A. My doctor is an avatar! The effect of anthropomorphism and emotional receptivity on individuals' intention to use digital-based healthcare services. Technol Forecast Soc Change. (2023) 191:122505. doi: 10.1016/j.techfore.2023.122505

[38] Canidate S Hart M. The use of avatar counseling for HIV/AIDS health education: the examination of self-identity in avatar preferences. J Med Internet Res. (2017) 19:e365. doi: 10.2196/jmir.6740, 29196281 PMC5732328

[39] Yu K Gorbachev G Eck U Pankratz F Navab N Roth D. Avatars for teleconsultation: effects of avatar embodiment techniques on user perception in 3d asymmetric telepresence. IEEE Trans Vis Comput Graph. (2021) 27:4129–39. doi: 10.1109/TVCG.2021.3106480, 34449373

[40] Li DD Liau AK Khoo A. Player–avatar identification in video gaming: concept and measurement. Comput Human Behav. (2013) 29:257–63. doi: 10.1016/j.chb.2012.09.002

[41] Van Looy J. Courtois C. De Vocht M.. 2010, Player identification in online games: validation of a scale for measuring identification in MMORPGs. In Proceedings of the 3rd International Conference on Fun and Games (pp. 126–134). ACM Leuven

[42] Lee HW Chang K Uhm JP Owiro E. How avatar identification affects enjoyment in the metaverse: the roles of avatar customization and social engagement. Cyberpsychol Behav Soc Netw. (2023) 26:255–62. doi: 10.1089/cyber.2022.0257, 37001178

[43] Klimmt C Hefner D Vorderer P. The video game experience as “true” identification: a theory of enjoyable alterations of players' self-perception. Commun Theory. (2009) 19:351–73. doi: 10.1111/j.1468-2885.2009.01347.x

[44] Klimmt C Hefner D Vorderer P Roth C Blake C. Identification with video game characters as automatic shift of self-perceptions. Media Psychol. (2010) 13:323–38. doi: 10.1080/15213269.2010.524911

[45] Sharma S. Global trends in virtual luxury: examining avatar identification, virtual materialism, and status consumption in online gaming. J Glob Mark. (2024) 37:282–303. doi: 10.1080/08911762.2024.2375709, 41277151

[46] Trepte S. Reinecke L. Effects of life-satisfaction, game competitiveness, and identification with the avatar. J Media Psychol. (2010) 22:171–184.

[47] Teng CI. Impact of avatar identification on online gamer loyalty: perspectives of social identity and social capital theories. Int J Inf Manag. (2017) 37:601–10. doi: 10.1016/j.ijinfomgt.2017.06.006

[48] Ko DW Park J. I am you, you are me: game character congruence with the ideal self. Internet Res. (2021) 31:613–34. doi: 10.1108/INTR-05-2020-0294, 35579975

[49] Rheu M Jang Y Peng W. Enhancing healthy behaviors through virtual self: a systematic review of health interventions using avatars. Games Health J. (2020) 9:85–94. doi: 10.1089/g4h.2018.0134, 31724888

[50] Mallmann GL Maçada ACG. The mediating role of social presence in the relationship between shadow IT usage and individual performance: a social presence theory perspective. Behav Inform Technol. (2021) 40:427–41. doi: 10.1080/0144929X.2019.1702100

[51] Short J Williams E Christie B. The social psychology of telecommunications. London: John Wiley & Sons (1976).

[52] Gao W Liu Z Li J. How does social presence influence SNS addiction? A belongingness theory perspective. Comput Human Behav. (2017) 77:347–55. doi: 10.1016/j.chb.2017.09.002

[53] Biocca F Harms C Burgoon JK. Toward a more robust theory and measure of social presence: review and suggested criteria. Pres Teleop Virt Environ. (2003) 12:456–80. doi: 10.1162/105474603322761270

[54] Mennecke B. E. Triplett J. L. Hassall L. M. Conde Z. J.. 2010). Embodied social presence theory. In 2010 43rd Hawaii international conference on system sciences (pp. 1–10). IEEE. Kauai, HI

[55] Garau M. Slater M. Vinayagamoorthy V. Brogni A. Steed A. Sasse M. A.. (2003). The impact of avatar realism and eye gaze control on perceived quality of communication in a shared immersive virtual environment. In Proceedings of the SIGCHI conference on Human factors in computing systems (pp. 529–536). Florida, USA: Association for Computing Machinery

[56] Nowak KL Biocca F. The effect of the agency and anthropomorphism on users' sense of telepresence, copresence, and social presence in virtual environments. Pres Teleop Virt Environ. (2003) 12:481–94. doi: 10.1162/105474603322761289

[57] Oh CS Bailenson JN Welch GF. A systematic review of social presence: definition, antecedents, and implications. Front Robot AI. (2018) 5:409295. doi: 10.3389/frobt.2018.00114, 33500993 PMC7805699

[58] Choi S. The flipside of ubiquitous connectivity enabled by smartphone-based social networking service: social presence and privacy concern. Comput Human Behav. (2016) 65:325–33. doi: 10.1016/j.chb.2016.08.039

[59] Kim DY Lee HK Chung K. Avatar-mediated experience in the metaverse: the impact of avatar realism on user-avatar relationship. J Retail Consum Serv. (2023) 73:103382. doi: 10.1016/j.jretconser.2023.103382

[60] Ochs M Bousquet J Pergandi JM Blache P. Multimodal behavioral cues analysis of the sense of presence and social presence during a social interaction with a virtual patient. Front Comput Sci. (2022) 4:746804. doi: 10.3389/fcomp.2022.746804

[61] Vilaro MJ Wilson-Howard DS Zalake MS Tavassoli F Lok BC Modave FP . Key changes to improve social presence of a virtual health assistant promoting colorectal cancer screening informed by a technology acceptance model. BMC Med Inform Decis Mak. (2021) 21:196. doi: 10.1186/s12911-021-01549-z, 34158046 PMC8218395

[62] Soutter ARB Hitchens M. The relationship between character identification and flow state within video games. Comput Human Behav. (2016) 55:1030–8. doi: 10.1016/j.chb.2015.11.012

[63] Anders SL Tucker JS. Adult attachment style, interpersonal communication competence, and social support. Pers Relat. (2000) 7:379–89. doi: 10.1111/j.1475-6811.2000.tb00023.x

[64] Kim J. Developing an instrument to measure social presence in distance higher education. Br J Educ Technol. (2011) 42:763–77. doi: 10.1111/j.1467-8535.2010.01107.x

[65] Coşkun S Bebiş H. Psychometric evaluation of a Turkısh version of the e-health literacy scale (e-heals) in adolescent. Gulhane Tip Derg. (2015) 57:378. doi: 10.5455/gulhane.157832

[66] Jackson SR Yu P Armany D Occhipinti S Chambers S Leslie S . Ehealth literacy in prostate cancer: a systematic review. Patient Educ Couns. (2024) 123:108193. doi: 10.1016/j.pec.2024.108193, 38354430

[67] Norman CD Skinner HA. eHEALS: the eHealth literacy scale. J Med Internet Res. (2006) 8:e507. doi: 10.2196/jmir.8.4.e27, 17213046 PMC1794004

[68] Berkman ND Davis TC McCormack L. Health literacy: what is it? J Health Commun. (2010) 15:9–19. doi: 10.1080/10810730.2010.499985, 20845189

[69] Edo OC Ang D Billakota P Ho JC. A zero trust architecture for health information systems. Health Technol. (2024) 14:189–99. doi: 10.1007/s12553-023-00809-4

[70] Wu S Zhang J Du L. “I do not trust health information shared by my parents”: credibility judgement of health (mis) information on social media in China. Health Commun. (2024) 39:96–106. doi: 10.1080/10410236.2022.2159143, 36548158

[71] Mbunge E Batani J Gaobotse G Muchemwa B. Virtual healthcare services and digital health technologies deployed during coronavirus disease 2019 (COVID-19) pandemic in South Africa: a systematic review. Global Health J. (2022) 6:102–13. doi: 10.1016/j.glohj.2022.03.001, 35282399 PMC8897959

[72] McElhinney E Kidd L Cheater FM. Health literacy practices in social virtual worlds and the influence on health behaviour. Glob Health Promot. (2018) 25:34–47. doi: 10.1177/175797591879333427466250

[73] Alshammari WMG Alshammari FMM Alshammry FMM. Factors influencing the adoption of e-health management among Saudi citizens with moderating role of e-health literacy. Inf Manag Bus Rev. (2021) 13:47–61. doi: 10.22610/imbr.v13i3(I).3251

[74] Kim K Shin S Kim S Lee E. The relation between eHealth literacy and health-related behaviors: systematic review and meta-analysis. J Med Internet Res. (2023) 25:e40778. doi: 10.2196/40778, 36716080 PMC9926349

[75] Shinbane JS Saxon LA. Virtual medicine: utilization of the advanced cardiac imaging patient avatar for procedural planning and facilitation. J Cardiovasc Comput Tomogr. (2018) 12:16–27. doi: 10.1016/j.jcct.2017.11.004, 29198733

[76] Jiang C Rashid RM Wang J. Investigating the role of social presence dimensions and information support on consumers' trust and shopping intentions. J Retail Consum Serv. (2019) 51:263–70. doi: 10.1016/j.jretconser.2019.06.007

[77] Jo E Lee SJ Han SH. Mediating effects of self-efficacy and social support on the relationship between eHealth literacy and self-care competency in patients undergoing percutaneous coronary interventions: a cross-sectional study. J Korean Acad Fundam Nurs. (2023) 30:325–34. doi: 10.7739/jkafn.2023.30.3.325

[78] Alviani R Purwandari B Eitiveni I Purwaningsih M. Factors affecting adoption of telemedicine for virtual healthcare services in Indonesia. J Inf Syst Eng Bus Intell. (2023) 9:47–69. doi: 10.20473/jisebi.9.1.47-69

[79] Oh Kruzic C Kruzic D Herrera F Bailenson J. Facial expressions contribute more than body movements to conversational outcomes in avatar-mediated virtual environments. Sci Rep. (2020) 10:20626. doi: 10.1038/s41598-020-76672-4, 33244081 PMC7692542

[80] Zempo K Yamazaki A Wakatsuki N Mizutani K Okada Y. Mouth-in-the-door: the effect of a sound image of an avatar intruding on personal space that deviates in position from the visual image. IEEE Access. (2022) 10:125772–91. doi: 10.1109/ACCESS.2022.3222804

[81] Ukaegbu O. C. Mingyue F. (2025). Examining the influence of personal eHealth literacy on continuance intention towards mobile health applications: A TAM-based approach. Health Policy and Technology, 101024.

[82] Sun S. Unlocking engagement: exploring the drivers of elderly participation in digital backfeeding through community education. Front Psychol. (2025) 16:1524373. doi: 10.3389/fpsyg.2025.1524373, 39981391 PMC11839607

[83] Zhang R Martí Casanovas M Bosch González M Sun S. Revitalizing heritage: the role of urban morphology in creating public value in China's historic districts. Land. (2024) 13:1919. doi: 10.3390/land13111919

[84] Lebel S Mutsaers B Tomei C Leclair CS Jones G Petricone-Westwood D . Health anxiety and illness-related fears across diverse chronic illnesses: a systematic review on conceptualization, measurement, prevalence, course, and correlates. PLoS One. (2020) 15:e0234124. doi: 10.1371/journal.pone.0234124, 32716932 PMC7384626

[85] Prettyman DV Bolls PD. The effects of sex-type, the sex of the avatar, and salience of the sex of the avatar on emotional valence and arousal. Front Psychol. (2021) 12:659547. doi: 10.3389/fpsyg.2021.659547, 34040567 PMC8141742

[86] Buetler KA Penalver-Andres J Özen Ö Ferriroli L Müri RM Cazzoli D . “Tricking the brain” using immersive virtual reality: modifying the self-perception over embodied avatar influences motor cortical excitability and action initiation. Front Hum Neurosci. (2022) 15:787487. doi: 10.3389/fnhum.2021.787487, 35221950 PMC8863605

[87] Craig TK Rus-Calafell M Ward T Leff JP Huckvale M Howarth E . Avatar therapy for auditory verbal hallucinations in people with psychosis: a single-blind, randomised controlled trial. Lancet Psychiatry. (2018) 5:31–40. doi: 10.1016/S2215-0366(17)30427-3, 29175276 PMC5746597

[88] Curtis RG Bartel B Ferguson T Blake HT Northcott C Virgara R . Improving user experience of virtual health assistants: scoping review. J Med Internet Res. (2021) 23:e31737. doi: 10.2196/31737, 34931997 PMC8734926

[89] Dimeff LA Jobes DA Chalker SA Piehl BM Duvivier LL Lok BC . A novel engagement of suicidality in the emergency department: virtual collaborative assessment and management of suicidality. Gen Hosp Psychiatry. (2020) 63:119–26. doi: 10.1016/j.genhosppsych.2018.05.005, 29934033

[90] Guo SHM Hsing HC Lin JL Lee CC. Relationships between mobile eHealth literacy, diabetes self-care, and glycemic outcomes in Taiwanese patients with type 2 diabetes: cross-sectional study. JMIR Mhealth Uhealth. (2021) 9:e18404. doi: 10.2196/18404, 33544088 PMC7895642

[91] Heidicker P. Langbehn E. Steinicke F. 2017). Influence of avatar appearance on presence in social VR. In 2017 IEEE symposium on 3D user interfaces (3DUI) (pp. 233–234) IEEE Los Angeles, CA

[92] Mason K Bicknell S Kreutzer E Wood A Hurlow J. Acceptability and feasibility of using avatar-based virtual world software as an adjunct to clinical interventions, training, and reflective practice in a medium secure setting: a qualitative interview study. Crim Behav Ment Health. (2022) 32:377–88. doi: 10.1002/cbm.2264, 36346206

[93] Moon J Kim E Choi S Sung Y. Keep the social in social media: the role of social interaction in avatar-based virtual shopping. J Interact Advert. (2013) 13:14–26. doi: 10.1080/15252019.2013.768051

[94] Ratan R Shen C Williams D. Men do not rule the world of tanks: negating the gender-performance gap in a spatial-action game by controlling for time played. Am Behav Sci. (2020) 64:1031–43. doi: 10.1177/0002764220919147

[95] Tan H Wei YC Yun HW Joan KEH Yee HW Juan LY. Health en eTM: developing a board game on value-based healthcare financing. Simul Gam. (2020) 51:87–105. doi: 10.1177/1046878119888710

[96] Triberti S Sebri V Savioni L Gorini A Pravettoni G. Avatars for clinical assessment: digital renditions of the self as innovative tools for assessment in mental health treatment In: Desjarlais M, editor. The psychology and dynamics behind social media interactions. Pennsylvania: Information Science Reference/IGI Global (2020)

[97] Turner JC Oakes PJ. The significance of the social identity concept for social psychology with reference to individualism, interactionism and social influence. Br J Soc Psychol. (1986) 25:237–52. doi: 10.1111/j.2044-8309.1986.tb00732.x

[98] Venkatesh V Thong JY Xu X. Consumer acceptance and use of information technology: extending the unified theory of acceptance and use of technology. MIS Q. (2012) 36:157–78. doi: 10.2307/41410412

[99] Voinea GD Gîrbacia F Postelnicu CC Duguleana M Antonya C Soica A . Study of social presence while interacting in metaverse with an augmented avatar during autonomous driving. Appl Sci. (2022) 12:11804. doi: 10.3390/app122211804

[100] Xu R Shi L Xia Y Wang D. Associations among eHealth literacy, social support, individual resilience, and emotional status in primary care providers during the outbreak of the SARS-CoV-2 Delta variant. Digit Health. (2022) 8:9789. doi: 10.1177/20552076221089789, 35355807 PMC8958311

[101] Short J Williams E Christie B. The social psychology of telecommunications. London: John Wiley & Sons. (1976).

[102] Animesh A Pinsonneault A Yang S-B Oh W. An odyssey into virtual worlds: exploring the impacts of technological and spatial environments on intention to purchase virtual products. MIS Quarterly (2011) 35:789–810. doi: 10.2307/23042799

[103] Andel SA Shen W Arvan ML Brown KG. Considering moral character in virtual reality: toward a virtue-based ethics of VR. Computers in Human Behavior. (2020) 106:106261.doi: 10.1016/j.chb.2019.106261

[104] Emmert M Wiener M. What factors determine patients’ satisfaction with hospital care? A literature review. International Journal for Quality in Health Care. (2017) 29:23–39. doi: 10.1093/intqhc/mzw152

[105] Liu P Shi J. Understanding users’ intention to use telehealth services: an empirical study integrating UTAUT and privacy calculus. Telemedicine and e-Health. (2021) 27:45–56. doi: 10.1089/tmj.2019.0305

